# Dental Education

**Published:** 1849-07

**Authors:** W. R. Handy


### ARTICLE VII.

### *Dental Education.* By W. R. HANDY, M. D.

IT is a gratifying fact, to be able to state, that *public attention* is now being fully aroused to the *importance of dental education*, and that already public opinion has expressed itself in a variety of ways, and in a manner not to be misunderstood—demanding at the hands of those who practice the dental art, that they shall first be thoroughly qualified, by receiving a proper and regular dental education.

In proof of this, it is only necessary to state, that in the South, where dental quackery seems to fatten and present a more than usual plethora, by appearing before its victims in a garb of most ample extent and pliancy, cut in such a form as not only to embrace every qualification, past and present, but still further, to leap into the future, by modestly asserting that all dentists, beside themselves, are strangely behind the age—and that they alone possess improvements, which are far, very far, ahead of the day—improvements which are absolutely essential to the beauty and durability of the teeth—such as have been long and fully tried and warranted—and such as they now, with all their bowels of benevolence and sympathy for suffering humanity, present to an intelligent public—assuring them, in all kindness and confidence, that all that is required on their part, is simply a trial to verify their assertions—and that, in the language of all such impostors, "No cure—no pay." By such flattering bait, the mouth was readily opened, the cure as readily applied, the pocket as readily and effectually sponged—and the victim so blindly and thoroughly duped, as not to make the discovery till too late to apply a remedy. Such, we say, was formerly the case at the South; but such presumptuous and unblushing pretensions are now by no means tolerated to the same extent as before; and though we have to admit that quackery still flourishes and thrives, it is nevertheless a gratification to know, that the credentials of this hydra-headed monster of dental notoriety are now demanded—and we are informed, upon the most reliable authority, that whenever and wherever educated men made their appearance—that then and there dental quackery became exposed, and had to "hide its diminished head;" in other words, when science and truth came in conflict with ignorance and error, that the people had intelligence sufficient to judge of the immense superiority of the former over the latter—that the former was as much for their health and happiness, as the latter was to entail disease and misery—and that, consequently, proper dental qualifications are now required, as essential passports to their confidence and support.

So much for the fact of public opinion in reference to the necessity of dental education. And why, or how, it may here be asked, has this change in public sentiment been brought about? The answer to this question brings us to the next point of consideration, viz. *the object of dental education.*

The *object* of dental education is assuredly the same as that of medical education, to wit: the relief of suffering humanity, by applying all the resources of medicine and surgery to the cure of the diseases of the dental organs—as that of medical education, under the head of general medicine and surgery, does to all the diseases of the whole body. In other words, dental education is only a link in the great chain of medical knowledge—that it is part and parcel of the same great science, rests upon the same immutable laws, and must consequently be studied in all its extent and relations, for the practical and proper application of those general principles which belong to any one part as well as to the whole of the body.

It is no objection to urge, that because the dental organs are of limited extent, that therefore the object is not the same—or at most, that dental education is not required to be near so extensive as that of the medical. With equal propriety, it might be urged, that as the eye and the ear form but very small portions of the whole body, that those, therefore, who devote their whole time to the cure of the diseases of these organs, have no need of any further knowledge of the rest of the system, and that, as they practice no further, that consequently their object is not the same as that of the general practitioner. But no—such a position has never for a moment been entertained by any one; but on the contrary, our ablest physicians and surgeons are always selected to preside over those large infirmaries for the cure of the diseases of the eye and the ear; and from the settled belief, and undoubted truth of such conviction, that he that is most familiar with the whole body and all its diseases, would be most likely to relieve any particular part of the same body, when deranged and requiring treatment. Now, if such be true of the eye and the ear—the same must be as certainly true of the teeth and the whole of the dental apparatus—for no one pretends to question the fact that the teeth are organized bodies, though this statement was formerly disputed, and even in this day of progressive knowledge and discovery, practically denied, by all the host of dental pretenders, who go to work upon the teeth as they would upon so many nails driven into a billet of wood, just as if there was not a spark of vitality present—though the sense of pain thrilling through the sensitive nerves of the patient, declares, in the loudest language of nature, that the teeth are organized, and that the assurances of boasted ignorance should stand abashed and be ashamed to attempt the repair of such delicate structures of nature's workmanship. But nature makes a further declaration of this organization of the teeth, by giving to them a definite birth, growth, development, decay and death, as to all the other organs, thus extending over the dental apparatus the same general laws of nutrition as the rest of the organism—which consequently brings the teeth under the same general influences disturbing the whole economy, and must, therefore, of necessity, require an equal extent of knowledge on the part of the dental operator to practice with the highest skill and success in his particular department, as for that of the surgeon in either of the special departments for the eye and the ear.

The dental student will thus perceive that proper dental qualifications, in the most comprehensive sense, are co-extensive with those of the medical, and that for his encouragement in this path of labor, he will find enrolled the brightest names of the dental profession—names whose examples he may be proud to follow, such as those of Parmly, Gardette, Harwood, Hullihen, Maynard, Foster, and many others of our own country, with Bell, Nasmyth, Koecker, Cartwright, Robinson, Tomes and others of Europe. These are some of the men who believed it necessary to master all the other departments of medicine, before they thought themselves thoroughly qualified to practice with the highest degree of efficiency any particular branch, even that of dentistry. With such men, dental physiology, and dental surgery, has made rapid strides, so much so, that the dental profession is now commanding equal respect, and claiming for itself equal honor and position in the great temple of the healing art.

To perpetuate and extend this high honor and use fulness is a sacred trust, bequeathed to those who are about or shall hereafter enter the ranks of the dental profession. To secure and guard this trust, we desire, therefore, most earnestly, to insist upon, as a fundamental element, the absolute necessity of a thorough dental education.

Presuming enough has been said in support of the position that the *object* in both a dental and medical education is the same—we are now prepared to discuss the last point we propose to notice, which is to consider the *means* of attaining this object.

The object of both professions being the same, it necessarily follows that the means used in each must also be the same—and these may be summarily arranged under three heads, viz.

1. Physical.
2. Mental.
3. Moral.

These threefold means or instrumentalities refer to the compound being or threefold nature of man, as being endowed with material instruments, intellectual, and moral powers.

The highest possible education of each of these several agencies, forms the surest guarantee of the highest possible skill, success, and eminence, both in the dental as well as the medical profession.

The *hands*, as the representatives of the physical means, must be constantly exercised, so as to acquire that nice tact so necessary, in all the various surgical and mechanical manipulations upon the mouth and face, and still further of unfolding, by means of the knife, all the various tissues of the body, so as to acquire that *anatomical knowledge*, which is of such vast importance as to form, at once, both the basis and guide in rearing the superstructures of either practical dentistry or general surgery—and this anatomical knowledge, so necessary for practical purposes, (we here desire to state,) can no where be obtained in the books—the dissecting room is the only place it is to be found, and can here be secured only by the most assiduous and untiring perseverance in the use of the knife, in dissecting the whole and every part of the body.

The *mental means* refer to the education of all the faculties of the mind, in the acquisition of all kinds of knowledge, both dental and medical, that can, in any way, assist the dental student in reaching the highest possible point of dental intellectual eminence, by which his reasoning powers will become so thoroughly drilled, that he can readily discriminate with the greatest conceivable accuracy the relation of cause and effect, and thus most effectually apply the vast and varied resources of his intellectual storehouse to the best method of restoring to health any of the dental organs that may be deranged.

The *moral means* constitute the master and finishing stroke of the dental education, which is deemed necessary to the attainment of the highest possible of professional dental excellence.

Without the education of the heart, in the exercise of all those high moral and elevated feelings of benevolence and charity, of obeying the golden rule of duty as well as of professional etiquette, to wit, of doing unto others as we would that others should do unto us. We say, without this latter species of education, all the brightness of the two former, however so brilliant, will necessarily wear a shadow, which in proportion to its extent and intensity, must, in like proportion eclipse the usefulness, and detract from the otherwise high eminence which has been attained.*

*If there is any one branch of medicine, the practice of which requires more honesty of purpose than another, it is the dental. With regard to the excellence of operations upon the teeth, no one but a competent practitioner can judge. The dentist has it in his power to practice the most barefaced imposition upon his patients without fear of immediate detection. He is often indirectly encouraged by the restlessness of his patient to do so. It is important, therefore, that the dentist should be honest as well as skillful.—*Balt. Ed.*

